# A study on how using an interactive multimedia e-book improves teachers’ ability to teach evidence-based medicine depending on their seniority

**DOI:** 10.1186/s12909-021-02984-2

**Published:** 2021-10-29

**Authors:** Yu-Hsuan Liao, Kuo-Shu Tang, Chih-Jen Chen, Ying-Hsien Huang, Mao-Meng Tiao

**Affiliations:** 1 Department of Pediatrics, Kaohsiung Chang Gung Memorial Hospital, Chang Gung University College of Medicine, Kaohsiung, Taiwan; 2 Chang Gung Medical Education Research Centre, CGMERC, No. 5, Fusing St., Gueishan Township, Taoyuan City, Taiwan; 3 Department of Clinical Education, Flinders University, Adelaide, Australia

**Keywords:** Evidenced-based medicine, e-books, Teaching

## Abstract

**Background:**

Teaching evidence-based medicine (EBM) is not an easy task. The role of the electronic book (e-book) is a useful supplement to traditional methods for improving skills. Our aim is to use an interactive e-book or PowerPoint to evaluate instructors’ teaching effects on EBM.

**Methods:**

Our study group was introduced to learning EBM using an interactive e-book available on the Internet, while the control group used a PowerPoint presentation. We adopted the Modified Fresno test to assess EBM skills both before and after their learning. EBM teaching sessions via e-book or PowerPoint were 20–30 min long, followed by students’ feedback. We adopted Student’s t-test to compare teachers’ evaluation of their EBM skills prior to the class and the students’ assessment of the teachers’ instruction. We also adopted repeated measures ANCOVA to compare teachers’ evaluation of their EBM skills using the Fresno test both before and after the class.

**Results:**

We observed no difference regarding EBM skills between the two groups prior to their experimental learning, which was assessed by the Modified Fresno test. After learning, physicians in the study group ranked higher in choosing a case to explain which kind of research design was used for the study type of the question and explaining their choice (P = 0.024) as assessed by the post-test to pre-test Fresno test. Teaching effect was better in the e-book group than in the control group for the items, “I am satisfied with this lesson,” “The teaching was of high quality,” “This was a good teaching method,” and “It aroused my interest in EBM.” However, no differences were observed between the two groups in physicians who had more than 10 years’ experience.

**Conclusions:**

The use of interactive e-books in clinical teaching can enhance a teacher’s EBM skills, though not in more senior physicians. This may suggest that teaching methodology and activities differ for teachers’ varying years of experience.

**Supplementary Information:**

The online version contains supplementary material available at 10.1186/s12909-021-02984-2.

## Background

Using the best evidence for determining a patient’s therapy is important [1]. Traditionally, the ability to make treatment decisions was limited to a physician’s own experience with the problem, which carries the potential risk of error [2–5]. The value of evidence-based medicine (EBM), which involves using updated, relevant, and trustworthy evidence to inform medical decisions, has been broadly acknowledged [6]. As a result, teaching EBM has become crucial for medical students’ development into lifelong independent learners and critical thinkers that can offer high-quality patient care [7]. The research problem is that physicians—even senior ones—may miss some of the important nuances of EBM, which subsequently impacts the quality of their teaching [7]. Given the lack of EBM teaching aids, we considered whether an interactive e-learning tool using SimMAGIC software [8] could be used as an effective teaching aid for EBM. In this study, our aim was to use an interactive e-book or PowerPoint to evaluate instructors’ teaching effects on EBM.

Chiu reported that, according to a national survey in Taiwan, the knowledge, skills, and attitudes regarding evidence-based practice (EBP) improved among physicians and nurses after undergoing training [9], thus indicating that the practice of EBM is important and can be improved through training [10]. Using Internet information sources to answer patient-related questions has taken an ever more important place in the daily practice of physicians [11]. A practical new instrument for measuring good-quality clinical application is vital for educating teachers and evaluating the effect of their teaching [12].

Electronic learning (e-learning) differs from traditional educational methods in that it uses digital platforms for learning [13, 14]. Using technology for learning has the benefit of being more student-focused and flexible [15]. The learner becomes a more active participant in the acquisition of knowledge, as opposed to being a passive recipient [13]. Cook reported that e-learning performs on par with traditional classroom teaching [16]. However, other researchers have reported that e-learning is no better than traditional classroom education for improving the proficiency of novices [17]. In modern curricula, e-book technology is considered a useful supplement to traditional methods [18, 19]. However, no studies have yet indicated whether the teaching effect of a lecturer is better after learning from an online e-book than traditional learning.

Our aim was to use an interactive e-book or PowerPoint to evaluate instructors’ teaching effects on EBM in order to evaluate the effectiveness of a clinical teaching EBM e-book to improve teacher-related skills, as well as to foster both teachers’ and learners’ interest in studying EBM. One month later, we arranged for every teacher to teach an EBM class and to have students assess the effectiveness of their teaching. This method may be a promising and effective way to improve physicians’ EBM-teaching skills in the future.

## Methods

### Ethical process

 This study was approved by the institutional review board of the ethics and clinical research committee of CGMH (104-9177B). All teachers and students provided their consent prior to participating in the study. To blind the statistician, the data was anonymized, and participants’ names were removed from the questionnaires before data analysis was performed. Data storage, participant recruitment, and data collection were all performed by a research assistant who had no assessment relationship with the participants.

### Study population

**Fig. 1:**
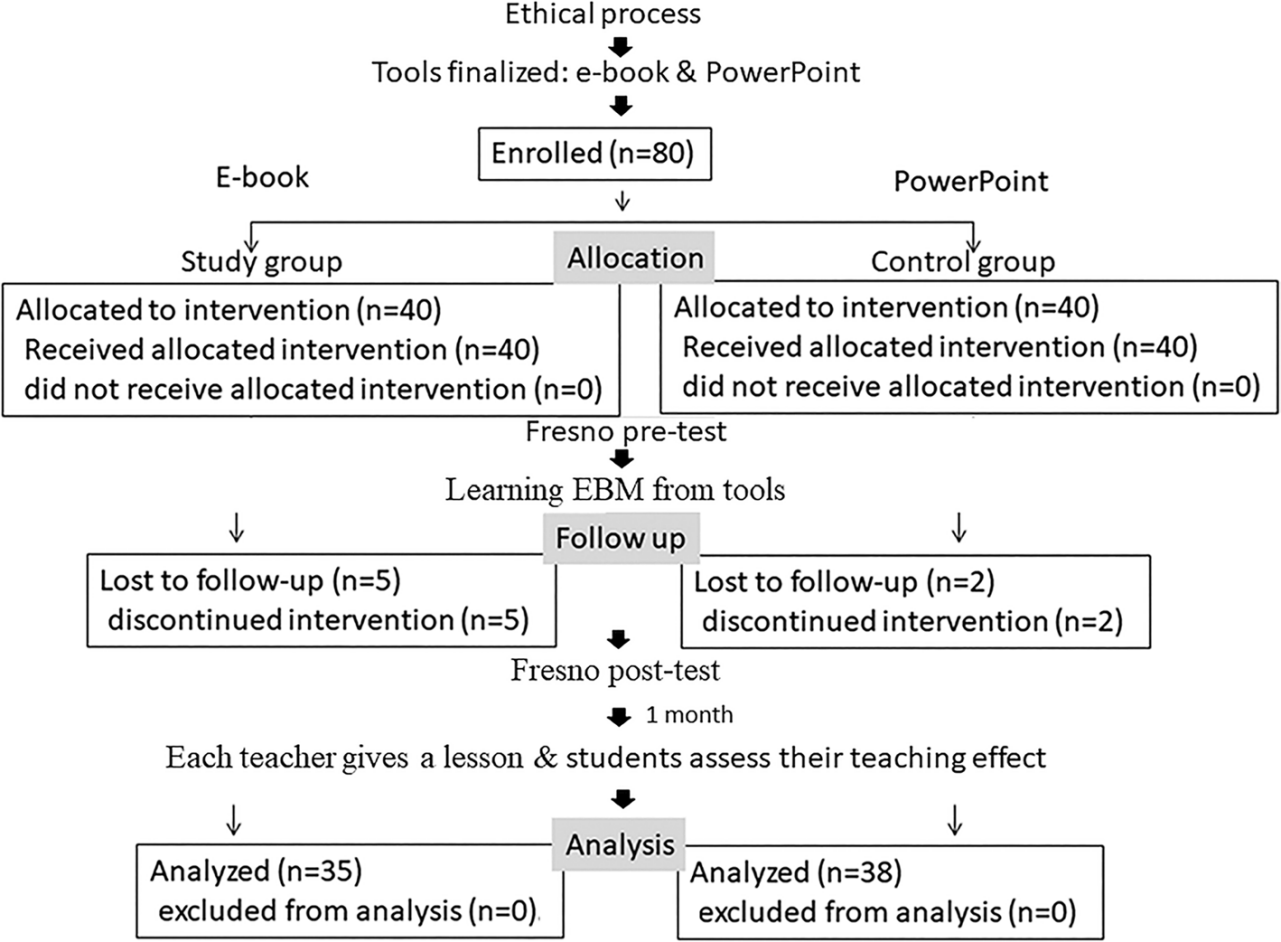
The procedures of the study and the flow diagram of the study selection of participants

This study was conducted from September 1, 2016 to October 31, 2017 (participants are shown in Table 1). Visiting staff teachers at Chang Gung Memorial Hospital (CGMH) were enrolled in this study, and two hundred teachers in the hospital were randomly selected by our group, choosing one for every 2.5 with the 500 names listed on our hospital’s computer. Those who did not consent to the assessment were excluded. The flow diagram of this study and its procedure is shown in Fig. 1. All teachers and students provided their consent before participation after understanding the study. A research assistant obtained the consent, and participants had the right to withdraw their data and consent at any time.

**Table 1:** Basic demographic characteristics of the teachers

Groups	e-book	PowerPoint
Total teachers (male %)	35 (48.6%)	38 (50.0%)
Departments		
Internal Medicine	9	10
Surgery	7	9
Pediatrics	7	8
Gynecology	6	6
Emergency	6	5
Years of experience		
< 5 years (male %)	13 (38.4%)	13 (46.2%)
–10 years (male %)	12 (50.0%)	14 (55.0%)
>10 years (male %)	10 (60.0%)	11 (54.5%)

### Tools: e-book or PowerPoint for teachers’ learning and teaching

Our team designed an online e-book using SimMAGIC software. The e-book contains an introduction to the basic concepts of EBM, EBM databases, database literature search skills, critical appraisal, and effectiveness evaluation methods using a repeatable answer-correct quiz [20, 21] (see availability of data and materials). The structure included EBM definition, EBM five steps, searching skills, RCT, systematic review, and four tests. The content for the e-book and PowerPoint was based on the EBM skills found on our hospital’s website at https://www1.cgmh.org.tw/library_s/EBMRdatabase.htm. (This cannot be accessed by Internet Explorer, but only Google Chrome, all rights reserved.)

A repeatable answer-correct quiz was provided to evaluate each of the learning objectives at the end of each topic. The learners can try the test multiple times to have better memory. Problem sets were created for readers and used for self-assessment purposes. They were presented in a multiple-choice question format, and readers could respond by clicking to answer the question. Another traditional method (PowerPoint) was designed for use as a control group (Additional file 1). The PowerPoint learning material was designed with the same contents as that of the e-book and also included the basic concepts of EBM, EBM databases, database literature search skills, critical appraisal methods, clinical application, and effectiveness evaluation. In the PowerPoint presentation, the questions were presented in a multiple-choice format but responses could not be provided by clicking.

### Study setting

In order to provide teachers with a better method of learning EBM and then assess their teaching effects through students’ feedback, a flipped classroom model was designed with an e-book or PowerPoint for teachers’ learning. In order to decrease the influence of students’ characteristics, the effect of teachers’ instruction was assessed by the same three participating 7th year students. It is very difficult to assess teachers’ day of the week and level of fatigue. Nevertheless, the study was appointed before teachers entered the study so they could answer the Fresno test and have a teaching class, thus minimizing the effect of other potentially confounding factors.

The teachers created an answerable question using the PICO model from a true case in the hospital and searched the electronic databases available at CGMH prior to the study. They attempted to consolidate the evidence in order to reach a conclusion and make a recommendation for the question. We then gave them access to the e-book or PowerPoint presentation and encouraged them to practice using it. They re-examined the information they had written by searching, consolidating evidence, and correcting the recommendation. Afterward, the assessment questionnaires shown in Table 2 were completed. The study group utilized the e-book, while the control group used the PowerPoint presentation without the e-book.

**Table 2:** Modified Fresno test in the e-book and PowerPoint groups (mean±SD)

Pre-test								
Questions					e-book (n=35)	PowerPoint (n=38)	p value	Effect size
Question 1. Write clinical questions based on context to search for articles to answer patient questions.					7.6±2.5	8.3±2.2	0.190	0.297
Question 2. Write three kinds of databases and explain their advantages and disadvantages.					8.0±2.5	8.0±2.3	0.289	0.000
Question 3. If you use PubMed or Medline to search for original papers, what is your search strategy? Include article categories and write the search techniques used to increase the search for the best article.					8.7±1.7	8.5±2.4	0.718	0.096
Question 4. Choose a case to explain which kind of research design is used for the study type of the question and explain your choice.					6.7±1.6	5.4±1.9	0.314	0.740
Question 5. What articles related to your problem were found? P: I: C: O:					9.0±2.0	8.6±2.6	0.552	0.172
Question 6. What are the research methods for the articles found, such as select patients, allocation, attrition, analysis, etc.?					8.9±1.9	8.6±2.4	0.847	0.138
Question 7. How are the important results expressed by the articles found, such as p-value, odds ratio or mean difference, 95% CI, effect size, etc.?					7.8±2.4	5.4±2.5	0.817	0.979
Question 8. There are articles describing the diagnosis of biliary obstruction and hepatitis patients; 200 patients have yellow gallbladder, 100 patients were later confirmed to have biliary obstruction, with 70 patients having an ultrasound gallbladder length less than 1.5 cm, and the ultrasound gallbladder length is less than 1.5 cm in 100 patients with hepatitis.					5.4±2.7	5.5±2.6	0.299	0.037
What is the sensitivity, specificity, positive predictive, negative predictive, and likelihood ratio in the diagnosis of biliary obstruction?								
Question 9. We find that in our hepatitis patients treated with ursodeoxycholic acid, who were followed for one year, 40% showed a decreased liver index (AST). In the group that was not treated with ursodeoxycholic acid, 20% showed a decreased liver index (AST).					6.2±3.9	5.3±4.0	0.110	0.227
I would like to ask: What is the absolute risk reduction? What is the relative risk reduction? What is the number needed to treat?								
Question 10. Children with diarrhea use probiotics. According to one report, the use of probiotics for unused probiotics reduces the number of diarrhea days, and the relative risk is 0.34 times. We would like to know if this result has statistical significance.					6.0±3.3	5.6±3.1	0.809	0.124
Please cite the number below that represents the confidence interval that indicates a statistical significance of the case: 0.1-0.6; 0.6-1.2; 1.2-2.1.								
Question 11. Which research design is the best for diagnostic research? RCT? Systematic review? Cohort study? Case control study? Others?					6.9±4.6	6.1±4.7	0.446	0.172
Question 12. Which study design is the best for prognostic research? RCT? Systematic review?					7.0±1.4	6.8±1.5	0.183	0.137
Cohort study? Case control study? Others?								
Total scores.					77.9±3.9	73.9±4.3	0.743	0.974
Post-test & post-test to pre-test difference								
	Post-test				Post-test to Pre-test			
Questions	e-book	PowerPoint	p value	Effect size	e-book difference	PowerPoint difference	p value	Effect size
	(n=35)	(n=38)						
Question 1. Write clinical questions based on context to search for articles to answer patient questions.	8.0±2.5	8.6±2.1	0.239	0.259	0.7±0.6	0.6±0.4	0.097	0.047
Question 2. Write three kinds of databases and explain their advantages and disadvantages.	8.0±2.5	8.7±2.1	0.239	0.303	0.7±0.5	2.1±0.6	0.963	0.000
Question 3. If you use PubMed or Medline to search for original papers, what is your search strategy? Include article categories and write the search techniques used to increase the search for the best article.	8.6±1.8	8.3±2.6	0.872	0.134	1.3±0.6	0.5±0.7	0.862	0.001
Question 4. Choose a case to explain which kind of research design is used for the study type of the question and explain your choice.	6.8±1.6	5.6±1.9	0.011*	0.683	1.4±0.5	0.2±0.4	0.024*	0.085
Question 5. What articles related to your problem were found? P: I: C: O:	9.1±1.9	8.9±2.5	0.886	0.09	-0.3±0.4	-0.1±0.6	0.365	0.014
Question 6. What are the research methods for the articles found, such as select patients, allocation, attrition, analysis, etc.?	8.8±2.1	8.5±2.6	0.737	0.126	2.0±0.9	1.8±0.8	0.790	0.001
Question 7. How are the important results expressed for the articles found, such as p-value, odds ratio or mean difference, 95% CI, effect size, etc.?	7.5±2.5	5.9±2.8	0.023*	0.602	0.8±0.6	-0.8±0.7	0.107	0.044
Question 8. There are articles describing the diagnosis of biliary obstruction and hepatitis patients; 200 patients have yellow gallbladder, 100 patients were later confirmed to have biliary obstruction, with 70 patients having an ultrasound gallbladder length less than 1.5 cm, and the ultrasound gallbladder length is less than 1.5 cm in 100 patients with hepatitis.	5.3±2.7	5.6±2.3	0.545	0.119	-0.8±0.9	0.2±0.6	0.563	0.006
What is the sensitivity, specificity, positive predictive, negative predictive, and likelihood ratio in the diagnosis of biliary obstruction?								
Question 9. We find that in our hepatitis patients treated with ursodeoxycholic acid, who were followed for one year, 40% showed a decreased liver index (AST). In the group that was not treated with ursodeoxycholic acid, 20% showed a decreased liver index (AST).	5.7±4.2	5.0±4.0	0.489	0.17	-0.5±1.0	0.4±0.8	0.152	0.035
I would like to ask: What is the absolute risk reduction? What is the relative risk reduction? What is the number needed to treat?								
Question 10. Children with diarrhea use probiotics. According to one report, the use of probiotics for unused probiotics reduces the number of diarrhea days, and the relative risk is 0.34 times. We would like to know if this result has statistical significance.	5.7±3.4	5.1±3.2	0.357	0.181	1.1±1.2	0.2±0.8	0.780	0.001
Please cite the number below that represents the confidence interval that indicates a statistical significance of the case: 0.1-0.6; 0.6-1.2; 1.2-2.1.								
Question 11. Which research design is the best for diagnostic research? RCT? Systematic review? Cohort study? Case control study? Others?	6.4±4.9	6.3±4.9	0.887	0.02	1.2±1.1	2.1±1.3	0.493	0.008
Question 12. Which study design is the best for prognostic research? RCT? Systematic review?	6.9±1.3	6.7±1.6	0.814	0.137	0.3±0.4	0.9±0.4	0.086	0.050
Cohort study? Case control study? Others?								
Total scores.	88.4±1.75	82.3±2.04	0.027*	2.999	10.5±4.6	8.4±3.7	0.122	0.041

*p<0.05, P: patient/problem, I: intervention, C: compare, O: outcome, AST: aspartate aminotransferase

A randomized controlled trial was established with a 1:1 allocation ratio to assess the teachers before they were assigned to either the e-book group (n = 40) or the control group (n = 40). Randomization was carried out (allocation concealment) through central randomization performed by an independent randomizer (assistant). Random assignment with a two-blocked design in the order of their entry into the study was adopted using http://www.randomizer.org. The teachers were blinded to the purpose of the study.

We employed an evaluation method using a structured paper questionnaire of the Modified Fresno test (Table 2) to investigate our teachers’ EBM skills both before and after the e-book or PowerPoint learning. The Fresno test questionnaire included three dimensions (knowledge, search abilities, and critical appraisal skills), with a total of 12 items (Table 2). The scale for each item was 0–10, the total scale for one Fresno test was 120. Teachers then practiced the EBM steps to search, critically appraise, and analyze the level of evidence for the article they found, make a recommendation, and apply it to a clinical situation.

One month later, each teacher gave a lesson on EBM according to the e-book (see availability of data and materials) or PowerPoint content shown in the S1 Appendix.

### Student assessment of the teachers’ EBM teaching effect

One month later, each teacher gave a 20 to 30-minute lesson on EBM regarding the formulation of problems, database search techniques, and literature appraisal for three students, who then assessed their teaching skills and ability. We provided eight statements for the three students for each teacher that they could answer with the following options: strongly agree, agree, neither agree nor disagree, disagree, and strongly disagree. We converted the scale into Likert scale with strongly agree 5, agree 4, neither agree nor disagree 3, disagree 2 and strongly disagree 1. The eight statements were as follows: “I am satisfied with this lesson,” “The teaching was of high quality,” “The teacher had a good attitude,” “This was a good teaching method,” “It helped my understanding,” “I was allowed to ask unlimited questions,” “The teacher listened to my questions,” and “It aroused my interest in EBM.”

### Validity

The content validity of the EBM skill questionnaire (Table 2) was examined by three experts who had no involvement with the participants and were asked to rate each item. The final survey included only items with strong relevance, which was defined in our previous papers [20–22]. The questionnaire had a content validity index of 0.90.

## Reliability

The internal consistency of all indexes was estimated using Cronbach’s α [23–25]. The questionnaire had a Cronbach’s coefficient α of 0.801.

### Statistical analysis

The sample size analysis was calculated using power analysis and sample size (G-Power) software with at least an effective sample size with 80 % power at the 5 % significance level (2-sided test) in each group for EBP, with the hypothesis of no difference between the two groups. Based on previous results (Chen CJ. Journal of Medical Education 2020) that showed an estimated standard deviation of 2.4 for the e-book group, respectively 2.5 for the PowerPoint group, a sample size of 16 teachers in each group would be able to detect a difference of 2.4 between the two groups. [21]. The evaluation of both EBM skills and teaching effects was analyzed as a continuous variable and expressed as mean ± standard error or mean difference. We adopted Student’s t-test to compare teachers’ evaluation of their EBM skills by Fresno test before the class, after the class and the students’ assessment of the teachers’ instruction. We adopted repeated measures ANCOVA to compare teachers’ evaluation of their EBM skills by Fresno test both before and after the class. The teachers were divided into sub-groups according to the length of their clinical experience: less than 5 years, 5–10 years, and more than 10 years of experience. A p-value < 0.05 was considered statistically significant. All statistical analyses were performed using Statistical Package for Social Science (SPSS, version 12) software.

## Results

### Participants

Two hundred teachers were assessed for eligibility, but 120 ultimately did not consent to the assessment due to being too busy with work. The 80 teachers who provided their consent were enrolled and randomly assigned to the e-book group (n = 40) or the traditional PowerPoint group (n = 40). Seven teachers were excluded for incomplete assessments. The final sample included 35 teachers in the e-book group and 38 in the PowerPoint group (Fig. 1). The basic demographic data of all the teachers are shown in Table 1.

### Analysis of the EBM skills of teachers with the pre- and post-Modified Fresno test

Prior to the class, the responses of each group assessed by the Modified Fresno test did not differ. After the class, repeated measures ANCOVA have been used in the second part of Table 2: the difference between the post-test value and the pre-test value for every teacher was then used to compare the two groups. The Modified Fresno test showed significant different total scores in the 12 questions between the e-book and PowerPoint groups (88.4 ± 1.75 vs. 82.3 ± 2.04, 95 %CI: 0.710-11.389, effect size = 2.999, p = 0.027). In particular, the fourth question about chose a case to explain which kind of research design was used for the study type of the question and explaining that choice (P = 0.011), and the seventh question about the important results expressed in the articles found (P = 0.023) significantly different between the two groups. (Table 2) But, only the fourth question differed significantly between the two groups compared the post-test to pre-test (P = 0.024) (Table 2).

## Teaching effects with feedback from students

**Fig. 2:**
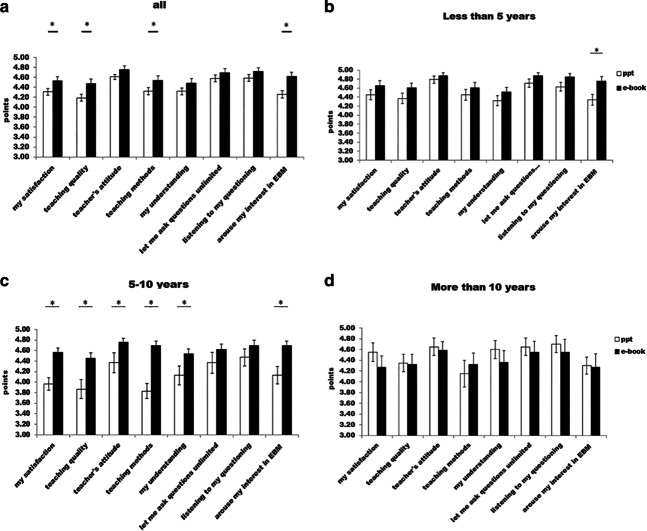
The effectiveness of the teaching between the e-book group and the PowerPoint group measured by the students of (**a**) all teachers, (**b**) physicians with less than 5 years’ clinical experience, (**c**) physicians with 5–10 years’ clinical experience, and (**d**) physicians with more than 10 years’ clinical experience. * P < 0.05

Teaching effects were better in the e-book group than in the PowerPoint group according to feedback from students with regard to four items. Those items were as follows: “I am satisfied with this lesson” (mean difference 0.226, P = 0.015), “The teaching was of high quality” (mean difference 0.285, P = 0.004), “This was a good teaching method” (mean difference 0.225, P = 0.027), and “It aroused my interest in EBM” (mean difference 0.358, P < 0.001) (Fig. 2a).

In the sub-group of physicians with less than 5 years’ experience, students’ feedback was better in “It aroused my interest in EBM” in the e-book group than in the PowerPoint group (difference 0.415, P = 0.007) (Fig. 2b).

In the physicians with 5–10 years’ experience, the students gave the e-book group higher ratings than the PowerPoint group for the following six items: “I am satisfied with this lesson” (difference 0.601, P < 0.001), “The teaching was of high quality” (difference 0.593, P = 0.001), “The teacher had a good attitude” (difference 0.390, P = 0.012), “This was a good teaching method” (difference 0.869, P < 0.001), “It helped my understanding” (difference 0.407, P = 0.012), and “It aroused my interest in EBM” (difference 0.569, P < 0.001) (Fig. 2c).

However, we observed no difference in students’ feedback for the physicians with more than 10 years’ experience between the two groups (Fig. 2d).

## Discussion

Improving teaching knowledge and problem-solving skills is important for teachers. Our study showed that the interactive e-book for EBM learning had better learning effects than those in the PowerPoint group according to the Modified Fresno test with regard to choose a case to explain which kind of research design was used for the study type of the question and explaining that choice. The interactive e-book learning improved the effectiveness of teaching in terms of greater satisfaction, better teaching quality, better teaching methods, and arousing students’ interest in EBM when compared to the traditional PowerPoint method. However, the groups with teachers who had less than 5 years’ experience and those with more than 10 years’ experience demonstrated fewer teaching differences.

The cause of the different learning and teaching effects regarding teachers’ seniority is not clear in this study, but may be related to the Taiwan Evidence-Based Medicine Association being established in 2007 [26]. We suppose that the different learning and teaching effects in the teachers’ seniority is the result of the promotion of EBM in Taiwan. One possible reason that physicians with 5–10 years’ experience are better at teaching is that the e-learning tutorial can provide an efficient and effective means of information delivery to junior doctors [27]. Therefore, they can share their experience of learning EBM, making it more interesting to their students.

The most commonly ranked barriers to the application of EBP include insufficient time, lack of skill in literature searching, and lack of skill in critical appraisal [28, 29]. In our study, the e-book group did not experience the problem of time, which offered significant improvement to the teachers because they could use it whenever convenient. The low rate of participation in our study may be due to physicians being too busy with work, and this may be improved for assessment on non-working days.

One previous study reported that approximately 30 % of residents did not complete their EBM exercises [30]. This finding indicates that a better teaching method is needed to improve teachers’ skills for students’ learning. Our interactive multimedia Internet e-book seems to meet this requirement. E-learning with structured materials provides predefined problem sets and has better student learning results [14, 31]. An e-learning approach to educating students to meet the criteria for evidence-based practice can result in higher-quality search strategies and improve confidence in EBM skills [32, 33]. The functions available in our e-book, combined with interactive quizzes and animations, resulted in improved learning effects in certain EBM skills.

In some cases, e-learning that uses e-books may have such challenges as cost and skill training [15], but our free Internet e-book does not have such problems. We used SimMAGIC to edit the e-book, which is simple and has a clear format. The cost of this software is inexpensive, and learners can use this e-book for free on the Internet [8]. Skills training in our e-book is not difficult, and as Persky has reported, an e-book is convenient in that it allows learners to pace themselves and have an active role in their learning [31]. Clear evidence from our data indicates that the teachers, especially those with 5–10 years’ experience, were able to extrapolate some of the knowledge gained from this practice.

## Conclusions

The use of interactive e-books in clinical teaching can enhance a teacher’s EBM skills, especially those with 5–10 years of clinical experience, though not in more senior physicians. This may suggest that teaching methodology and activities differ for teachers’ varying years of experience.

## Limitations

This study has certain limitations that are worth noting. Since it is a single-site study that uses a specifically designed instrument, our findings need to be tested in a large-scale study for generalizability to other populations in different locations. The low rate of participation in our study is another limitation that needs further consideration when interpreting its results.

## Supplementary Information

**Additional file 1.** The PowerPoint for the control group.

## Data Availability

The Modified Fresno test and students’ feedback data during the current study are available from the corresponding author upon reasonable request. The e-book is available at https://ebooks.hamastar.com.tw/Upload/ebook/20413/Html5/index.html. After downloading SilverLight_x64, go to the web address via Windows Internet Explorer (it is not available via Google Chrome).

## Abbreviations

EBM: evidence-based medicine
e-book: electronic book
EBP: evidence-based practice
e-learning: electronic learning
