# Supplementary material for: A study on how using an interactive multimedia e-book improves teachers’ ability to teach evidence-based medicine depending on their seniority
Source: BMC Med Educ. 2021 Oct 29;21:547. doi: 10.1186/s12909-021-02984-2 (PMC8555285; doi:10.1186/s12909-021-02984-2)
Supplement: Supplementary file 1 — Additional file 1. The PowerPoint for the control group. [file 12909_2021_2984_MOESM1_ESM.pdf]

實 證 醫 學

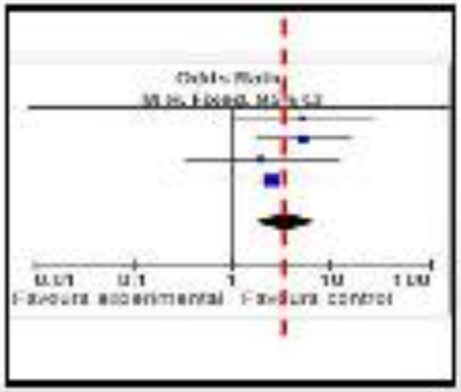

刁茂盟製作

高雄長庚紀念醫院

目錄

1. EBM definition
2. EBM 5 steps
3. Searching skills
4. RCT
5. Systematic review
6. test 1
7. test 2
8. test 3
9. test 4

Evidence-based medicine (EBM) : defintion

1. the conscientious, explicit and judicious
2. use of current best evidence in making decisions
3. about the care of individual patients

- Conscientious: careful to do everything
- explicit : express clearly and directly
- Judicious: wise: done sensibly and carefully

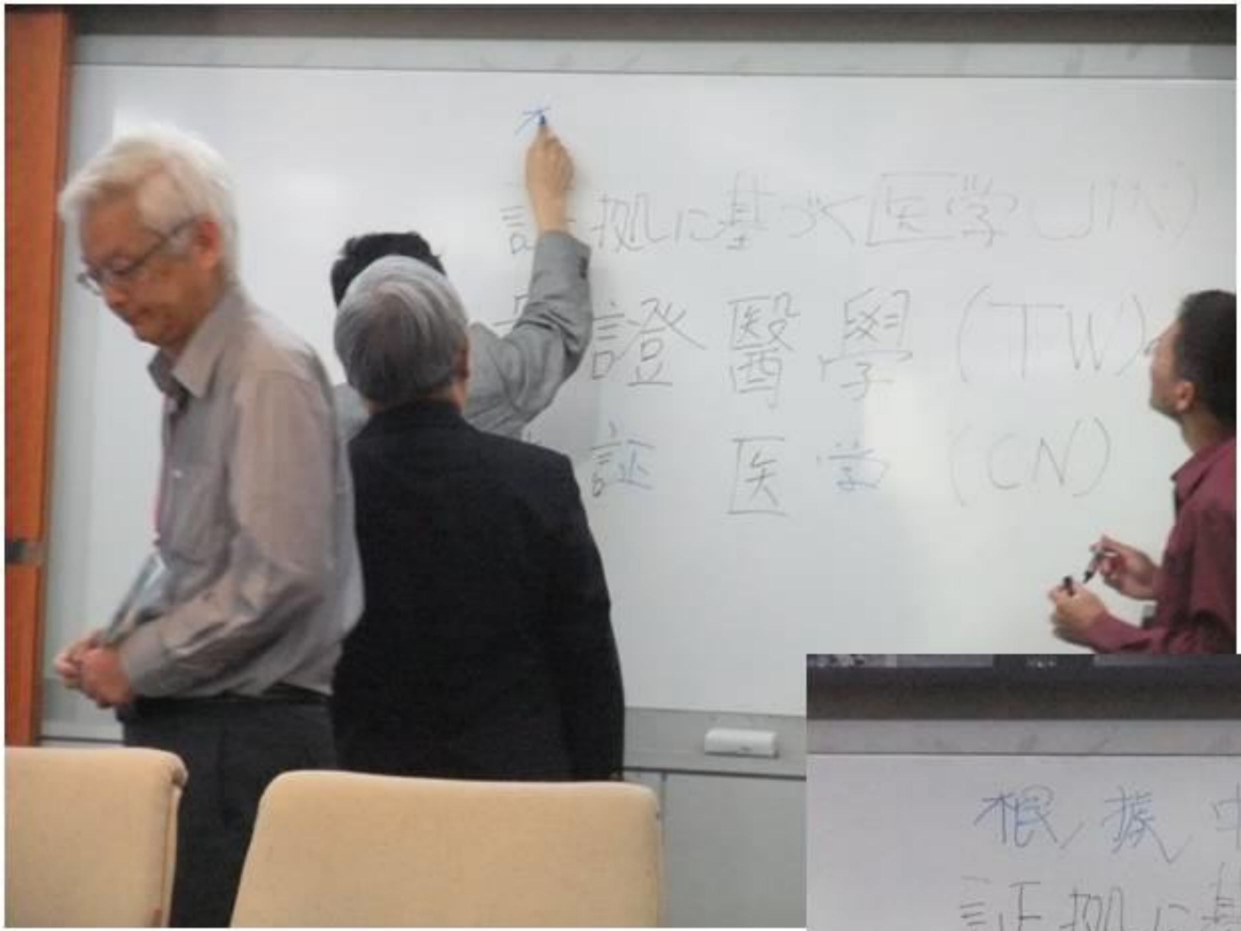

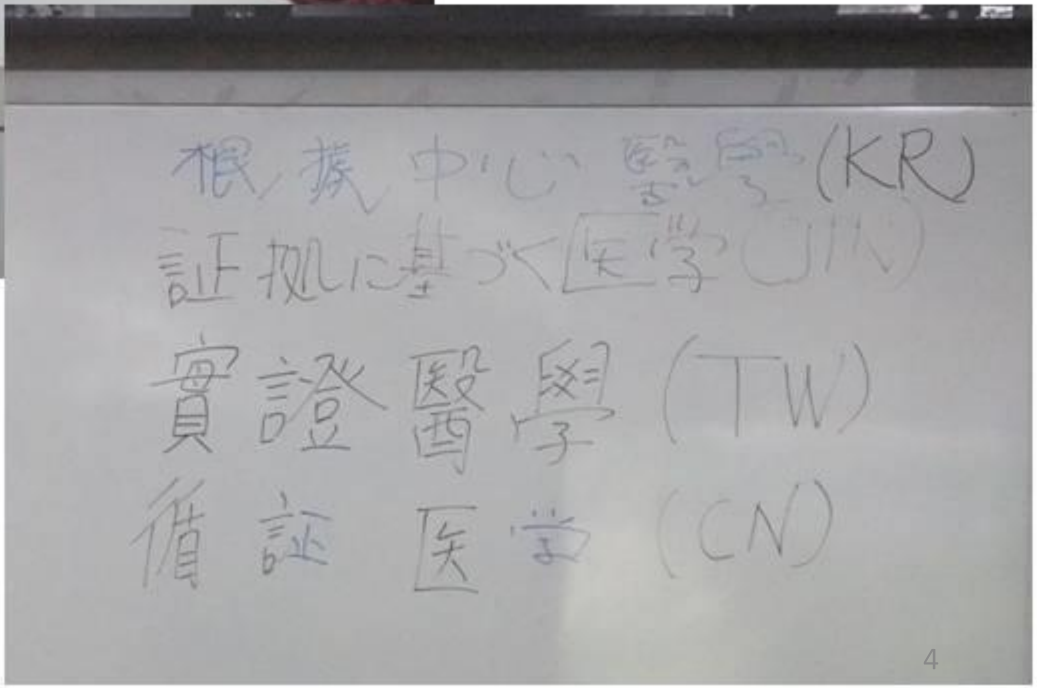
根據中心醫學 (KR)
証拠に基づく医学
實證醫學 (TW)
循証医学 (CN)

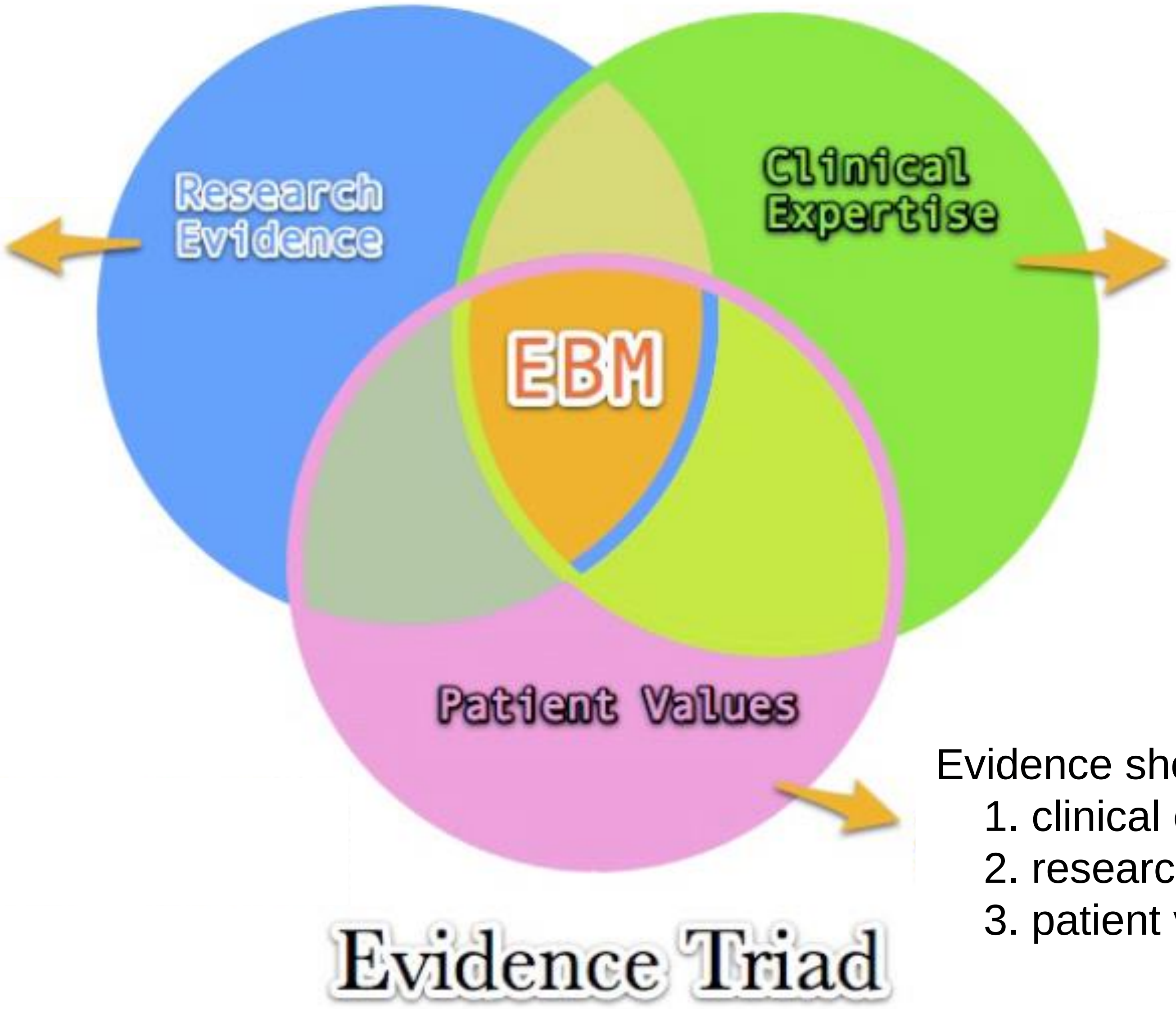

Evidence should be included

1. clinical expertise
2. research evidence
3. patient value

EBM 5 steps

1. Asking: Converting the clinical uncertainties into an answerable question.
2. Accessing: Search the database and tracking down the best available evidence
3. Appraising: Critical appraising that evidence for its validity and importance
4. Applying: Integrating the critical appraising with our clinical expertise and our patient’s unique biology, values and circumstances
5. Auditing: Evaluating our effectiveness and efficiency in executing step 1- 4 and seeking ways to improve them both for next time

From: http://www.hsl.unc.edu/services/tutorials/ebm_searching/pages/intro.htm

Type of Question	Suggested best type of Study
Therapy	RCT>cohort > case control > case series
Diagnosis	prospective, blind comparison to a gold standard
Etiology/Harm	RCT > cohort > case control > case series
Prognosis	cohort study > case control > case series
Prevention	RCT>cohort study > case control > case series
Clinical Exam	prospective, blind comparison to gold standard
Cost	economic analysis

Step 1: asking: Answerable question

PICO

- Patient and/or Problem
- Intervention/ or Exposure
- Comparison intervention (if relevant)
- Outcomes

Step 2: Accessing: Searching skills

- Systematic retrieval of the best evidence available

1. Identify terms to fit your PICO question
2. Look for secondary sources:
   – UpToDate, Cochrane, EBMR, DynaMed…
3. Search for primary sources
   – PubMed, Medline, nursing reference, EMBASEUsing, OVIDYou…

Step 3 Appraising

- Randomization: the two (or more) groups of subjects are followed in exactly the same way
- the only differences in terms of procedures, tests, outpatient visits, and follow-up calls
- Follow up: <20% loss is better
- Blind: procedures that prevent study participants, caregivers, or outcome assessors from knowing which intervention was received
  - "single-blind," "double-blind," and "triple-blind“
  - participants, care providers, those assessing outcomes

Step 4 Applying
Categories of recommendations

- **Level A: Good scientific evidence suggests that the benefits of the clinical service substantially outweigh the potential risks.**
- **Level B: At least fair scientific evidence suggests that the benefits of the clinical service outweighs the potential risks.**
- **Level C: At least fair scientific evidence suggests that there are benefits provided by the clinical service, but the balance between benefits and risks are too close for making general recommendations.**
- **Level D: At least fair scientific evidence suggests that the risks of the clinical service outweighs potential benefits. Clinicians should not routinely offer.**
- **Level I: Scientific evidence is lacking, of poor quality, or conflicting, such that the risk versus benefit balance cannot be assessed.**

Step 5 auditing

1. Am I asking any clinical questions at all?
2. Am I asking well-formulated questions:
   - ✓ Two-part questions about "background" knowledge?
   - ✓ Four- (or three-) part questions about "foreground" diagnosis, management, etc.?
3. Am I using a "map" to locate my knowledge gaps and articulate questions?
4. Can I get myself "unstuck" when asking questions?
5. Do I have a working method to save my questions for later answering?

(SE Straus et al. 2005: Evidence-based medicine – how to practice & teach EBM, 3rd ed.)

Randomized controlled trial

- specific type of scientific experiment, and the gold standard for a clinical trial
- used to test the efficacy or effectiveness of various types of medical intervention within a patient population
- May provide an opportunity to gather useful information about adverse effects, such as drug reactions

http://en.wikipedia.org/wiki/Randomized_controlled_trial

Number needed to treat / harm (NNT/NNH)

- ways of expressing the effectiveness and safety of an intervention in a way that is clinically meaningful.
- In general, computed two treatments A and B
- A typically a drug; and B a placebo
- probabilities %A and %B of treatments A and B
- NNT = 1/(%B-%A)
- example, an NNT of 4 means if 4 patients are treated, only one would respond.

Systematic review

- A **systematic review** is a literature review
- focused on a research question that tries to identify, appraise, select and synthesize all high quality research evidence relevant to that question.
- Systematic reviews of high-quality randomized controlled trials are crucial to evidence-based medicine

Meta-analysis

- Meta-analyses are often, but not always, important components of a systematic review procedure
- methods that focus on contrasting and combining results from different studies
- hope of identifying patterns among study results, sources of disagreement among those results, or other interesting relationships
- more powerfully estimate the true effect size as opposed to a less precise effect size derived in a single study
- a framework called estimation statistics which relies on effect sizes, confidence intervals and precision planning to guide data analysis, and is an alternative to null hypothesis significance testing.

http://en.wikipedia.org/wiki/Meta-analysis

EBM

Q: 5 steps of evidence based medicine?

1	2	3

4	5

apply	apprasial	ask	Assess	audit
應用證據	評讀	提問	搜尋	評估

Q: Is the follow up enough？
How many % completed the study and this make the study reliable?

1. 80%
2. 70%
3. 50%

Q: Can statin reduce subarachnoid hemorrhage?

take statin 100 persons with 7 got subarachnoid hemorrhage, control with 100 persons and 17 got subarachnoid hemorrhage (incidence of delayed ischemic deficits)

0.07	0.1	0.4
0.17	9	10

relative risk(RR)=

absolute risk reduction (ARR)=

number need to treat (NNT) =

relative risk
RR=(7/100)/(17/100)=0.41

absolute risk reduction=
17/100-7/100=10/100=0.1

NNT =
1/ (17/100-7/100)=1/0.1=10

Q:Homogeneity and heterogeneity?

Study or Subgroup	Experimental Events	Experimental Total	Control Events	Control Total	Weight	Odds Ratio M-H, Fixed, 95% CI
Asikainen 2002b	5	100	2	200	12.4%	5.21 [0.99, 27.35]
Braith RW 1994	10	200	5	500	26.7%	5.21 [1.76, 15.44]
Duncan JJ 1991	3	300	2	400	16.7%	2.01 [0.33, 12.11]
Hamdorf 1992	25	1000	3	300	44.2%	2.54 [0.76, 8.47]
Total (95% CI)		1600		1400	100.0%	3.50 [1.75, 6.98]
Total events	43		12			

Heterogeneity: $Chi^2 = 1.38$, $df = 3$ ($P = 0.71$); $I^2 = 0\%$

Test for overall effect: $Z = 3.55$ ($P = 0.0004$)

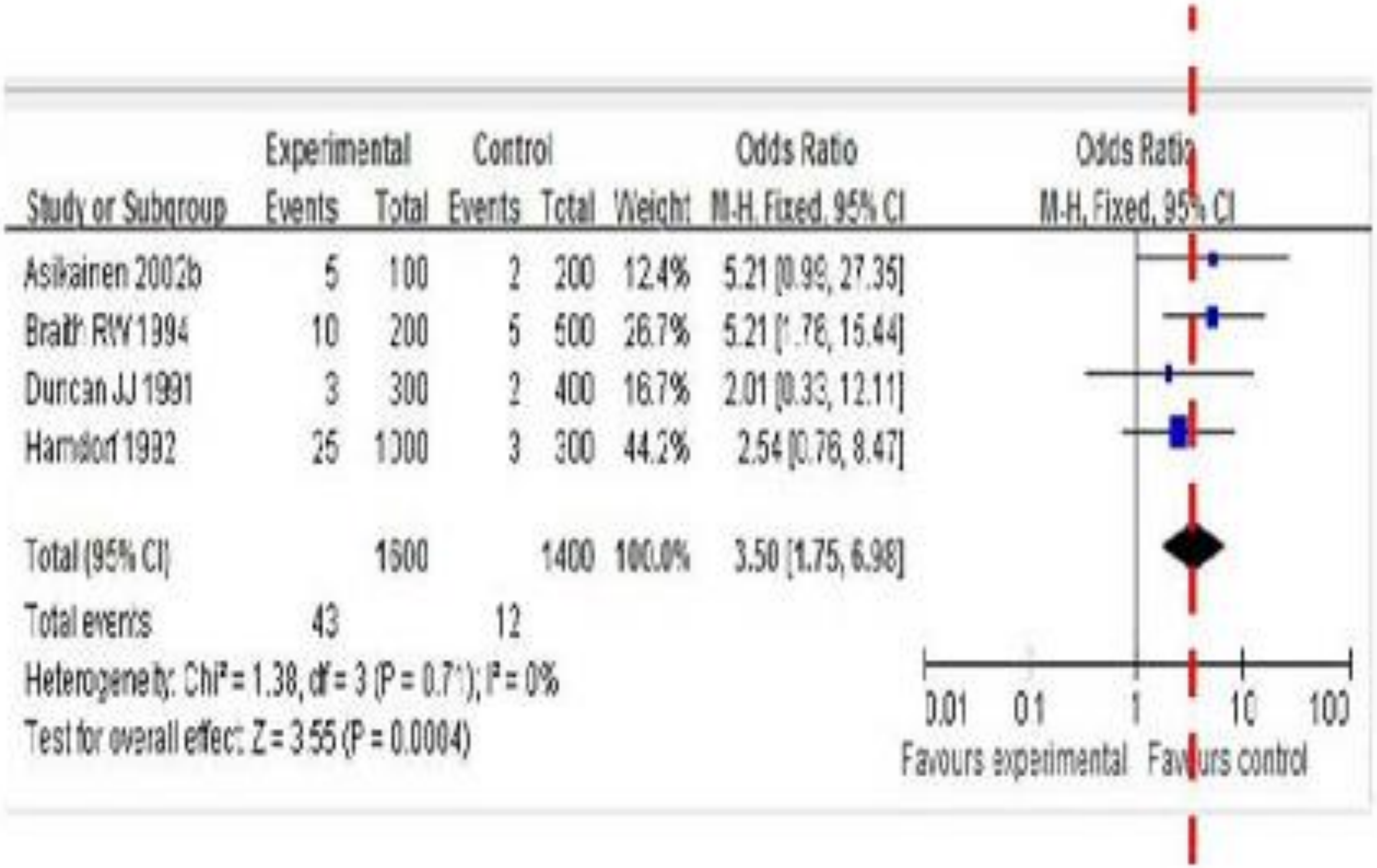

Q:homogenity and heterogeneity?

Heterogeneity: $Chi^2 = 1.38$, $df = 3$ ($P = 0.71$); $I^2 = 0\%$
Test for overall effect: $Z = 3.55$ ($P = 0.0004$)

Heterogenity p=

Q=

Q/df=

I^2=

<0.1	>0.1
>0.05	0%
< 1	>1
1.38	4%
3.55	

2001 Oxford Center for EBM: Levels of Evidence

建議等級	證據標準	Therapy/Prevention/Etiology/Harm
A	1a	系統性回顧 Systematic review<br>(分析數個隨機臨床對照試驗, 其結果均類似)
A	1b	設計良好, 結果精確 之隨機(RCT)臨床對照試驗
A	1c	All or none
B	2a	系統性回顧(SR) (分析數個世代研究, 其結果均類似)
B	2b	世代研究 Cohort study; 設計粗糙之隨機臨床對照試驗 (<50%RCT)
B	2c	"Outcomes" Research; Ecological studies
B	3a	系統性回顧 (分析數個病例 - 對照研究, 其結果均類似)
B	3b	病例 - 對照研究 Individual Case-control study
C	4	某家醫院的十年經驗; 設計不良之世代研究 及病例 - 對照研究
D	5	未經考證之專家個人意見, 基礎研究, 細胞實驗, 生理實驗, 動物實驗…的結果

Level of evidence 2001

Oxford Centre for Evidence-based Medicine Levels of Evidence (May 2001)

Level	Therapy/Prevention, Aetiology/Harm	Prognosis	Diagnosis	Differential diagnosis/symptom prevalence study	Economic and decision analyses
1a	SR (with homogeneity*) of RCTs	SR (with homogeneity*) of inception cohort studies; CDR† validated in different populations	SR (with homogeneity*) of Level 1 diagnostic studies; CDR† with 1b studies from different clinical centres	SR (with homogeneity*) of prospective cohort studies	SR (with homogeneity*) of Level 1 economic studies
1b	Individual RCT (with narrow Confidence Interval‡)	Individual inception cohort study with ≥ 80% follow-up; CDR† validated in a single population	Validating** cohort study with good††† reference standards; or CDR† tested within one clinical centre	Prospective cohort study with good follow-up****	Analysis based on clinically sensible costs or alternatives; systematic review(s) of the evidence; and including multi-way sensitivity analyses
1c	All or none§	All or none case-series	Absolute SpPins and SnNouts††	All or none case-series	Absolute better-value or worse-value analyses ††††
2a	SR (with homogeneity*) of cohort studies	SR (with homogeneity*) of either retrospective cohort studies or untreated control groups in RCTs	SR (with homogeneity*) of Level >2 diagnostic studies	SR (with homogeneity*) of 2b and better studies	SR (with homogeneity*) of Level >2 economic studies
2b	Individual cohort study (including low quality RCT; e.g., <80% follow-up)	Retrospective cohort study or follow-up of untreated control patients in an RCT; Derivation of CDR† or validated on split-sample§§§ only	Exploratory** cohort study with good†††reference standards; CDR† after derivation, or validated only on split-sample§§§ or databases	Retrospective cohort study, or poor follow-up	Analysis based on clinically sensible costs or alternatives; limited review(s) of the evidence, or single studies; and including multi-way sensitivity analyses
2c	"Outcomes" Research; Ecological studies	"Outcomes" Research		Ecological studies	Audit or outcomes research
3a	SR (with homogeneity*) of case-control studies		SR (with homogeneity*) of 3b and better studies	SR (with homogeneity*) of 3b and better studies	SR (with homogeneity*) of 3b and better studies
3b	Individual Case-Control Study		Non-consecutive study; or without consistently applied reference standards	Non-consecutive cohort study, or very limited population	Analysis based on limited alternatives or costs, poor quality estimates of data, but including sensitivity analyses incorporating clinically sensible variations.
4	Case-series (and poor quality cohort and case-control studies§§)	Case-series (and poor quality prognostic cohort studies***)	Case-control study, poor or non-independent reference standard	Case-series or superseded reference standards	Analysis with no sensitivity analysis
5	Expert opinion without explicit critical appraisal, or based on physiology, bench research or "first principles"	Expert opinion without explicit critical appraisal, or based on physiology, bench research or "first principles"	Expert opinion without explicit critical appraisal, or based on physiology, bench research or "first principles"	Expert opinion without explicit critical appraisal, or based on physiology, bench research or "first principles"	Expert opinion without explicit critical appraisal, or based on economic theory or "first principles"

Produced by Bob Phillips, Chris Ball, Dave Sackett, Doug Badenoch, Sharon Straus, Brian Haynes, Martin Dawes since November 1998.

Oxford Centre for Evidence-Based Medicine 2011 Levels of Evidence

Oxford **2011**

Question	Step 1 (Level 1*)	Step 2 (Level 2*)	Step 3 (Level 3*)	Step 4 (Level 4*)	Step 5 (Level 5)
**How common is the problem?** common	Local and current random sample surveys (or censuses)	Systematic review of surveys that allow matching to local circumstances**	Local non-random sample**	Case-series**	n/a
**Is this diagnostic or monitoring test accurate?** (Diagnosis)	Systematic review of cross sectional studies with consistently applied reference standard and blinding	Individual cross sectional studies with consistently applied reference standard and blinding	Non-consecutive studies, or studies without consistently applied reference standards**	Case-control studies, or "poor or non-independent reference standard**	Mechanism-based reasoning
**What will happen if we do not add a therapy?** (Prognosis)	Systematic review of inception cohort studies	Inception cohort studies	Cohort study or control arm of randomized trial*	Case-series or case-control studies, or poor quality prognostic cohort study**	n/a
**Does this intervention help?** (Treatment Benefits)	Systematic review of randomized trials or *n*-of-1 trials	Randomized trial or observational study with dramatic effect	Non-randomized controlled cohort/follow-up study**	Case-series, case-control studies, or historically controlled studies**	Mechanism-based reasoning
**What are the COMMON harms?** (Treatment Harms)	Systematic review of randomized trials, systematic review of nested case-control studies, *n*-of-1 trial with the patient you are raising the question about, or observational study with dramatic effect	Individual randomized trial or (exceptionally) observational study with dramatic effect	Non-randomized controlled cohort/follow-up study (post-marketing surveillance) provided there are sufficient numbers to rule out a common harm. (For long-term harms the duration of follow-up must be sufficient.)**	Case-series, case-control, or historically controlled studies**	Mechanism-based reasoning
**What are the RARE harms?** (Treatment Harms)	Systematic review of randomized trials or *n*-of-1 trial	Randomized trial or (exceptionally) observational study with dramatic effect			
**Is this (early detection) test worthwhile?** (Screening)	Systematic review of randomized trials	Randomized trial	Non -randomized controlled cohort/follow-up study**	Case-series, case-control, or historically controlled studies**	Mechanism-based reasoning

Impact of quality of evidence on strength of recommendation

GRADE system

(Grading of Recommendations Assessment, Development and Evaluation)

Find out

Judge to compare

4. Write the recommendation and determine the strength (strong or weak/conditional)

Determine whether the recommendation is "for" or "against" an intervention.

- If benefits outweigh downsides, guideline panels will recommend "for" an intervention.
- If downsides outweigh benefits, guideline panels will recommend "against" an intervention.

Determine the wording of recommendation. There are 3 wording options.

	Wording 1	Wording 2	Wording 3
Strong recommendation for	We recommend...	Clinicians should...	We recommend...
Weak recommendation for	We suggest	Clinicians might...	We conditionally recommend...
Weak recommendation against	We suggest...not	Clinicians might not...	We conditionally recommend...not
Strong recommendation against	We recommend ...not	Clinicians should not...	We recommend ...not

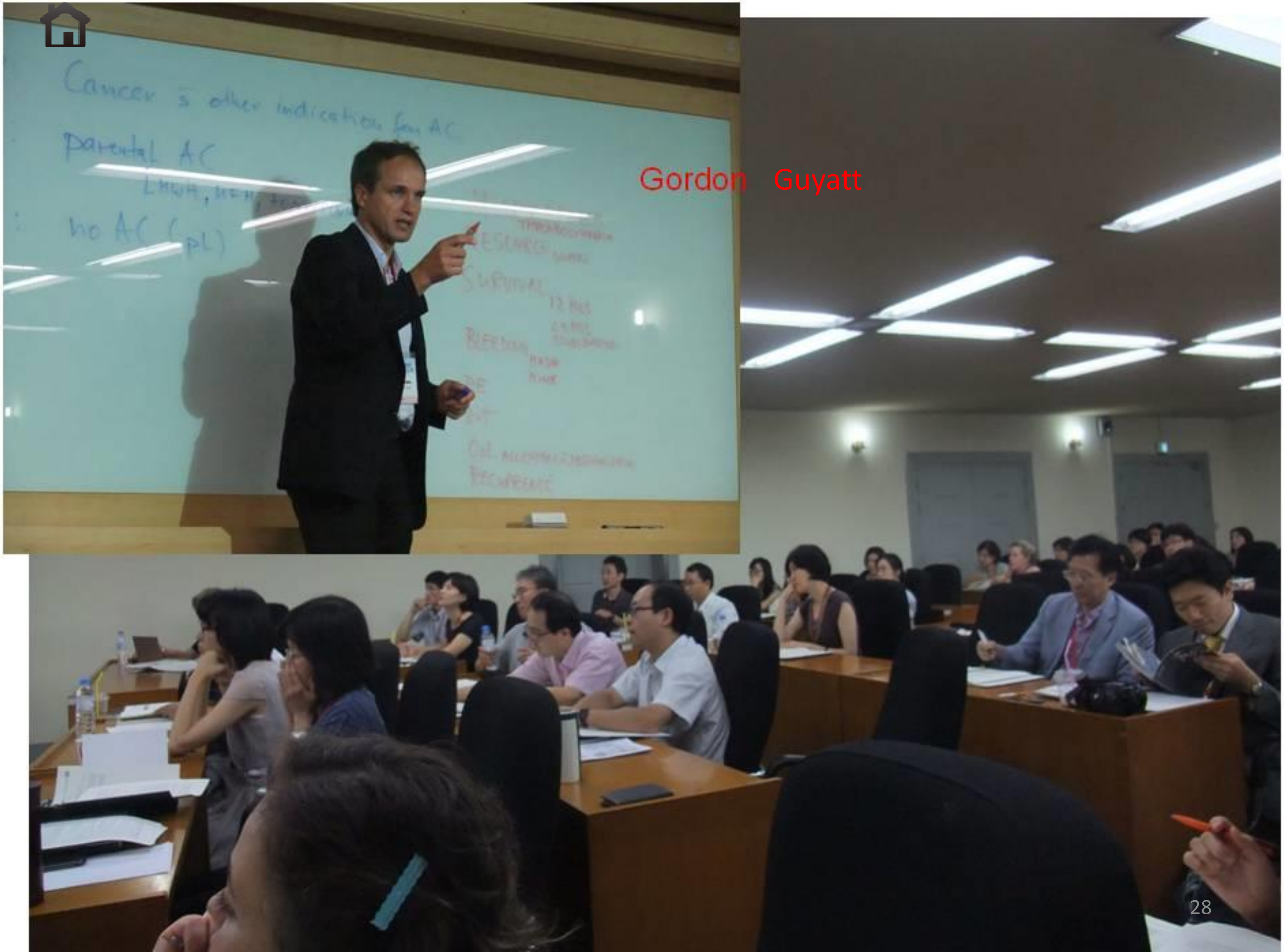

Gordon Guyatt

GRADE guidelines: 4 domains, 5 quality, 6 bias

1.Quality of evidence

1. Limitations of design
   – bias
2. Inconsistency
   – Larger dose for sicker
3. Indirectness
   – Different PIC to the (O)outcome
4. Imprecision
   – Sample size, 95%CI
5. Publication bias

→

1. Allocation concealment
2. Blinding
3. Loss of follow – up
4. Intention to treat
5. Stop early
6. Neglect to report outcomes

2.Balance of benefits vs harms & burdens

3.Values and preferences

4.Resource implications

GRADE guidelines: Four domains

一. Quality of evidence
- Key reasons for downgrading or upgrading
- Whether data for outcome is not available?

二. Balance of benefits versus harms and burdens
- Baseline risks for benefit or harms
- Modeling require?

三. Values and preferences
- Perspective 均衡taken and source
- Methods determine values satisfactory?

四. Resource implications
- Cost per unit, feasibility, opportunity costs, settings
